# Effects of Dietary Lysolecithin Supplementation during Late Gestation and Lactation on Sow Reproductive Performance, Sow Blood Metabolic Parameters and Piglet Performance

**DOI:** 10.3390/ani12050623

**Published:** 2022-03-01

**Authors:** Georgios A. Papadopoulos, Alexandra L. Wealleans, Georgios A. Delis, Geert P. J. Janssens, Mauro di Benedetto, Paschalis Fortomaris

**Affiliations:** 1 Laboratory of Animal Husbandry, School of Health Sciences, Faculty of Veterinary Medicine, Aristotle University of Thessaloniki, 541 24 Thessaloniki, Greece; fortomap@vet.auth.gr; 2 Kemin Animal Nutrition & Health, Kemin Europa N.V., Toekomstlaan 42, 2200 Herentals, Belgium; alexandra.wealleans@kemin.com (A.L.W.); mauro.dibenedetto@kemin.com (M.d.B.); 3 Laboratory of Pharmacology, School of Health Sciences, Faculty of Veterinary Medicine, Aristotle University of Thessaloniki, 541 24 Thessaloniki, Greece; delis@vet.auth.gr; 4 Department of Nutrition, Genetics and Ethology, Faculty of Veterinary Medicine, Ghent University, 9820 Merelbeke, Belgium; geert.janssens@ugent.be

**Keywords:** sows, lysolecithin, backfat, lactation, litter weight

## Abstract

**Simple Summary:**

Lactation is metabolically very demanding, and sows struggle to eat enough to cover their requirements. Use of lysolecithin can improve energy digestibility and retention and may be able to help sows maintain condition and performance during lactation. In the present study supplementation with lysolecithin reduced backfat loss and increased litter growth. There were also impacts of lysolecithin supplementation on glucose and urea metabolism, indicating improved nutrient digestion and absorption, potentially via leptin-regulated mechanisms.

**Abstract:**

The objective of the present study was to evaluate the effects of dietary supplementation of lysolecithin in sows’ diets during the last three weeks of the gestation period and throughout the lactation period on performance and metabolic parameters. In total 60 sows were allocated to two treatments: (a) CG (control group): the sows were fed commercially control diets; (b) LLG (lysolecithin group): the sows were fed the control diets supplemented with 750 g/t of feed supplemented with lysolecithin (Lysoforte Booster Dry^TM^, Kemin Europa N.V., Herentals, Belgium). Backfat was lower in LLG than CG at end of gestation and at weaning (*p* = 0.030 and 0.044, respectively), while the CG sows mobilized more backfat between day 14 to weaning (*p* = 0.006). Litter weight at weaning was higher in the LLG (*p* = 0.027). Fasted glucose levels at day 14 of lactation tended to be lower in LLG compared to CG (*p* = 0.074). Urea concentrations were higher in LLG than CG at day 14 (*p* = 0.002). Lysolecithin supplemented sows compared to the control mobilized less tissue during lactation to support lactation demands. In conclusion, lysolecithin supplementation in sows resulted in improved litter weight at weaning without an excessive catabolism of backfat tissue, most probably due to an efficient nutrient utilization, which warrants further investigation.

## 1. Introduction

The modern, intensive pig industry requires high-performing sows that are also very demanding. Over the years sows have become more prolific and produce large, heavy litters with increased daily gains and high requirements for energy consumption from milk [1,2,3]. These fast-growing litters impose an energy deficit on the sow, especially when entering a catabolic state towards the end of the gestation and early lactation [4]. Commonly, sows are fed a restricted diet shortly before, and for the first days after farrowing to prevent decreased lactation feed intake or postpartum dysgalactia syndrome [5,6]. Therefore, increasing energy and nutrient retention from feed is crucial to maintain performance while keeping feed costs reasonable [2,6]; the addition of animal fats, such as tallow or lard, can help nutritionists achieve these aims [3]. Despite their high energy density, however, animal fats can be highly variable in their composition and quality [7] and are associated with comparatively incomplete absorption, since they mainly contain saturated, long-chain fatty acids (FAs). These saturated long-chain fatty acids (LCFAs) are known to be less readily incorporated in the micelles of the intestinal lumen [8,9], a prerequisite for exposure to the activity of pancreatic lipases [10,11].

Addition of digestive enzymes and emulsifiers within the feed of both sows and fattening pigs has also been used to aid in digestion, absorption, and retention of fat and other nutrients [9,12]. Lysolecithin (LL) is one of the most studied emulsifying agents added to improve digestibility [13]. Usually produced by processing soybean oil [14,15], commercially available LLs contain diverse mixtures of phospholipids (PLs), lysophospholipids (LPLs), triglycerides (TGs) and other compounds [16]. It has been reported that LL emulsifying properties vary depending on its source and dietary fat composition, essentially on the ratio of saturated vs. unsaturated FAs [8,13,17,18], though this is debated when the wider effects of husbandry are considered [19]. At the same time, LL can interact with the structure and function of biological membranes, further enhancing nutrient absorption by the intestinal epithelial cells [14,16].

The sensitive performance and metabolic balance of sows on transition from gestation to lactation and the possible positive effect of LL supplementation has been a topic of research. Zhao et al. [20] studied the effect of LL addition (beginning on gestation day 110) to a low-animal fat or a high-animal fat diet given to gestating/lactating sows on their performance, litter weight gain, milk composition and total tract digestibility of energy and nutrients, while Wang et al. [21] also explored the impact of linearly increasing levels of the same as above LL-containing product (administered from gestation day 107) on performance and digestibility indices. They furthermore studied the respective impact on milk/colostrum immunoglobulin concentrations and on selected blood parameters. However, the feed did not contain animal fat and sows were allowed ad libitum consumption during the entire lactation period.

Overall, it could be hypothesized that dietary supplementation of lysolecithin during the demanding period of the last part of gestation and throughout lactation in sows could affect their metabolism and performance by improving digestibility and absorption of fat as well as other nutrients. In this context, the objective of the study was to of LL supplementation on an animal fat-containing diet from three weeks before expected farrowing and during the lactation period, on sow and litter performance and on selected blood metabolism associated parameters in sows.

## 2. Materials and Methods

### 2.1. Animals, Housing, and Experimentation Feeding Protocol

The animal experiment was performed in a commercial farrow-to-finish operating pig farm, with a capacity of 740 sows, located within the Pieria Regional Unit (Kontariotisa area), Region of Central Macedonia, Greece. In total, sixty (*n* = 60) multiparous Topigs sows were used for the conduct of this study. On gestation day 90, whilst kept in solid concrete floor gestation pens, they were randomly allocated into two equally sized groups, which were managed under the same feeding regime. Specifically, sows belonging to the first, control (CG) group (*n* = 30; mean parity: 4.40) or to the second experimental group (LLG) (*n* = 30, mean parity 4.76) received the same standard corn/barley/wheat-based gestation and lactation diets (Table 1). The diets of the second (LLG) group were supplemented “on top” with a commercially available LL-containing product (LYSOFORTE™ Booster Dry; Kemin Europa N.V., Herentals, Belgium) at 750 mg/kg of feed. From gestation day 90 up to gestation day 108 (one week before the expected farrowing) sows were provided 3.5 kg of the gestation diet.

On day 108, sows were transferred to the farrowing rooms and daily dietary provision was switched to 3.5 kg of the lactation diet. On gestation day 113 (two days before the expected farrowing) the daily quantity of feed provided was reduced to 1.5 kg and on the farrowing day to 1 kg. Rations were gradually increased from the next day onwards up to a maximum of 2 kg + 0.4 kg per piglet. Cross-fostering was practiced, if necessary, within the sows of the same treatment and during the first 24 h postpartum. The experimental timeline ended at weaning, on lactation day 28. Sow feed intake was monitored daily and any significant refusals of more than 0.5 kg/day were also recorded. Maximum feed quantity during lactation was reached at the sixth day post-farrowing. Daily feed quantity during the lactation was provided to sows in three different meals (08.00, 13.00 and 17.00 h). Feed was offered manually. It should be noted that there were no differences between treatments regarding the feed consumption either during the gestation or the lactation period.

### 2.2. Measurements of Performance Parameters and Blood Sampling

Measured sow performance parameters included: (i) body weight (measured on gestation day 108 and at weaning, both after an overnight fast), (ii) backfat thickness (measured on gestation day 108, on lactation day 14 and at weaning), (iii) weaning-to-estrus interval. Backfat thickness was measured at the P2 position (6.5 cm to the left and right from the dorsal midline at the level of the last rib) on standing sows, ultrasonically, with use of a Renco LEAN-MEATER^®^ (Renco Corp., Minneapolis, MN, USA). Derived parameters included: (i) body weight loss from gestation day 108 to weaning (absolute and relative), and (ii) backfat thickness loss from the backfat difference between gestation day 108 to lactation day 14, from gestation day 108 to weaning, and from lactation day 14 to weaning. Furthermore, based on Papadopoulos et al. [2] the backfat loss percentage (expressed in %) was calculated for the aforementioned intervals following the equation: Backfat loss % = [(Backfat-previous measurement − Backfat-later measurement)/Backfat-previous measurement] × 100.

Piglet performance parameters, measured on farrowing day and at weaning comprised: (i) liveborn piglets after cross fostering, (ii) litter weight at birth after cross fostering, (iii) number of weaned piglets, and (iv) litter weight at weaning. The rest derived parameters were: (i) number of piglets lost during lactation per litter, (ii) average individual weight at birth, (iii) coefficient of variation of piglet weight at birth, (iv) average individual weight at weaning, (v) coefficient of variation of piglet weight at weaning.

Blood samples were collected from sows on gestation day 108 and on lactation day 14. On each occasion, both groups (CG and LLG) were randomly divided into two equally sized subgroups, namely A and B (*n* = 15). Blood droplets were collected from the animals of subgroups A after performing a small incision at their tail base before the morning meal and at 30, 60, 90 and 120 min post-prandially. Collected blood droplets were inserted in Accutrend^®^ Plus system portable analyzers (F. Hoffmann-La Roche AG, Basel, Switzerland) for determination of glucose (GLU) and triglycerides levels. Heparinized BD Vacutainer^®^ blood collection tubes (Becton, Dickinson and Company; Franklin Lakes, NJ, USA) were used for blood plasma harvest from the sows of subgroups B, 60 min after finishing their morning meal, to be used for the quantitative determination of urea, non-esterified fatty acids (NEFAs), creatinine and leptin as previously described [2]. Leptin was determined using a commercially available RIA test kit (Multi-Species Leptin RIA Kit^®^, catalogue number XL- 85K; Linco Research, Inc., St Charles, MO, USA). Creatinine, urea and NEFA were measured spectrophotometrically (Ultrospec IIE, LKB, Biochrom, Cambridge, UK) using a commercial colorimetric diagnostic kit (Randox Laboratories, Crumlin, UK).

### 2.3. Statistical Analysis

All analyses were performed using the SPSS v. 25.0 software (IBM Corp., Chicago, IL, USA). Individual sows and litters (*n* = 30 per treatment) were set as the experimental units. Blood parameters were compared between treatments from samples collected from two equally sized subgroups (*n* = 15 per treatment). Performance and blood parameters following the normal distribution and displaying equality of variance among groups were analyzed with One-way Analysis of Variance (ANOVA), groups being used as the fixed factor. Additionally, data on glucose and triglycerides and gestation day 110 and lactation day 14 were analyzed with repeated measures analysis of General Linear Model procedure of SPSS. Level of significance was always set at 5 %, whereas a *p* value between 0.05 and 0.1 was considered as indicative of a statistical trend [8]. The Area Under the concentration-time Curve (AUC) for glucose and triglycerides evaluated at different timepoints pre- and post-prandially was calculated according to the trapezoidal rule with the use of the specific function GraphPad Prism (version 9.1.2 for Windows^®^, GraphPad Software, San Diego, CA, USA).

## 3. Results

The effects of treatment on the performance parameters of sows and piglets are summarized in Table 2 and Table 3. Bodyweights differed neither on gestation day 108 nor at weaning. Bodyweight loss during those five weeks rose to almost a quarter of their initial weight. Sows of the CG group had a significantly *(p* = 0.030) thicker backfat layer than those of the LLG group at d108 and d14; however, the difference in backfat thickness between the groups gradually disappeared towards weaning. Particularly between d14 and weaning, the backfat loss was smaller in LLG than in CG (*p* = 0.006). The degree of backfat tissue mobilization did not affect their post-weaning reproductive performance, as sows of both groups came to estrus soon and almost concurrently: 5.83 vs. 6.03 d after weaning, for groups CG and LLG, respectively (*p* = 0.834).

Litter size did not differ between the groups. Losses due to mortality during lactation were within acceptable criteria and comparable between groups. Litter weights were similar at birth, but litters of sows whose feed was LL-supplemented arrived significantly heavier at weaning (*p* = 0.027).

**Figure 1:**
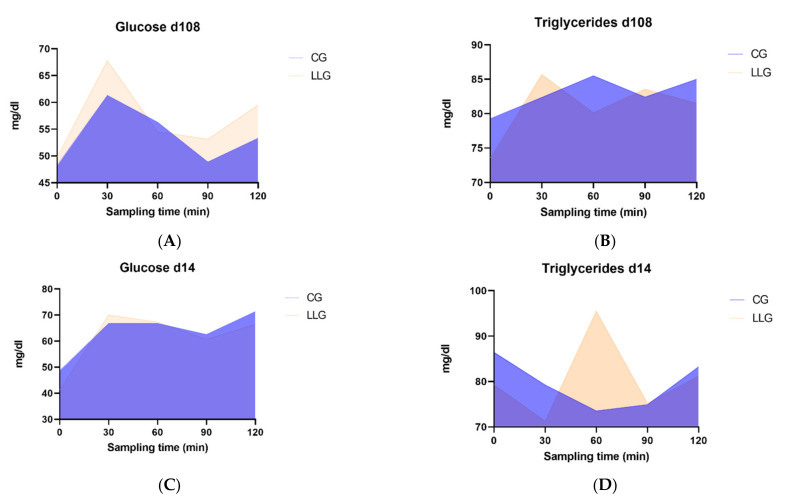
Areas under the curve (AUC) for the glucose and triglycerides levels which were measured pre- (0 min) and post- (30, 60, 90, 120 min) prandially in sows fed the control diet (CG group) or the control diet supplemented with lysolecithin at 750 g/t (LLG group) (*n* = 15 per treatment). According to the analysis, at day 108 of gestation the total AUC for glucose levels was 6521 for CG and 6902 for LLG, while the peak value in both groups occurred at 30 min postprandially and was 61.33 mg/dL and 67.79 mg/dL for CG and LLG group, respectively (panel **A**). For triglycerides levels at day 108 the total AUC was 9972 for CG and 9809 for LLG, while the peak value occurred for CG at 60 min (85.50 mg/dL) and for LLG at 30 min (85.7 mg/dL) (panel **B**). At day 14 of lactation the total AUC for glucose levels was 7687 for CG and 7557 for LLG, while the peak value occurred for CG at 120 min (71.36 mg/dL) and earlier for LLG at 30 min (70.00 mg/dL) (panel **C**). At day 14 the total AUC for triglycerides levels was 9377 for CG and 9671 for LLG, and the peak value was detected for CG at 0 min (86.42 mg/dL) and for LLG at 60 min (95.54 mg/dL) (panel **D**).

The concentration-time profiles of glucose and triglycerides in blood following the morning meal on gestation day 108 and on lactation day 14 are given in Table 4 and Table 5, respectively, while the AUC are provided in Figure 1. Although data from gestation day 108 revealed no differences in glucose between the two groups, pre-prandial levels on lactation day 14 showed a trend (*p* = 0.074) to be lower in the LLG animals. No differences were discerned between the treatments at any time point, on any day for the triglyceride concentrations. Repeated measures analysis of glucose and triglycerides levels collected at the end of gestation showed no significant treatment effect (*p* = 0.262 for glucose and *p* = 0.225 for triglycerides), no significant interaction between treatment and time of sampling (*p* = 0.626 for glucose and *p* = 0.492 for triglycerides) and a significant time effect (*p* < 0.001 for glucose and *p* = 0.044 for triglycerides). Moreover, repeated measures analysis of glucose and triglycerides levels collected at mid-lactation showed no significant treatment effect (*p* = 0.420 for glucose and *p* = 0.851 for triglycerides), no significant interaction between treatment and time of sampling (*p* = 0.583 for glucose and *p* = 0.247 for triglycerides) and a significant time effect for glucose (*p* < 0.001) but not for triglycerides (*p* = 0.247).

On gestation day 108, differences in urea NEFA and creatinine blood concentrations between the two groups were not statistically significant (Table 6). Leptin concentration was significantly higher (*p* = 0.021) in the animals of the CG group compared to LLG. This was not the case on lactation day 14, when differences in leptin, along with NEFA and creatinine, were non-significant between CG and LLG groups. On the contrary, urea levels were significantly (*p* = 0.002) elevated in the animals of group LLG.

## 4. Discussion

Sows during the last third of gestation have dramatically increased needs for dietary energy [3,20]. Two thirds of total fetal growth occur within this period, uterine blood flow rises, and the development of insulin resistance leads to an increase in blood glucose levels and fetal glucose uptake. Lactate and NEFA blood levels are also elevated towards the end of gestation, indicating a negative energy balance and mobilization of fat (and protein) reserves, even in cases where ad libitum feeding is allowed [4]. Subsequent milk production is also highly energetically demanding, and means sows need both a suitable dietary energy supply and improved digestion, absorption, and retention processes. The present study explored how LL supplementation throughout the last third of gestation and the entire lactation period could support performance.

According to our results, LL did not significantly affect the body weight of sows at either time point. Backfat thickness of sows belonging to the LLG group was lower than that of sows of the CG group at gestation day 108 and lactation day 14, implying a similar peripartal loss rate; however, this was not the case at weaning, since animals of both groups had similar backfat reserves, probably signifying a reduction in body fat loss during the last half of lactation due to the addition of LL in the feed. Indeed, it was revealed that sows in the LLG group mobilized less backfat than CG sows during the last half of the lactation period. The second half of the lactation period is the period with the highest metabolic demands for the sow, because milk production is maximized as well as piglet growth. A lower backfat mobilization during this crucial period, implies that the sows in the LLG group may have been able to tolerate the energy needs, by not only mobilizing body reserves, but most probably by an improved digestion and metabolic efficiency of nutrients due to lysolecithin supplementation. In the study of Laws et al. [22] sows supplemented with palm oil, lost less backfat than those who were not supplemented. The reduction in backfat loss was attributed to increased dietary energy intake during lactation. Elsewhere, supplementing various levels of soybean lecithin oil in lactating sows’ diets, did not affect backfat mobilization during lactation [23]. In the latter study, backfat was measured at farrowing and at weaning, and therefore no indication of backfat mobilization during the mid-lactation could be noted. In accordance with our findings, lysophospholipid supplementation in sows from day 107 of gestation onwards, resulted in a reduction of body weight and backfat loss during lactation [21]. This effect was probably mediated by an improved availability of nutrients due to the action of lysophospholipid. It is plausible that lysolecithin also improved digestibility and availability of nutrients in the present study. Such an effect could be supported by the increased urea levels in the LLG group during lactation, which may suggest an improved utilization of absorbed amino acids, irrespective of lean body mass mobilization, since that would have led to a change in blood creatinine. Whereas it is commonly considered that the primary function of amino acids from dietary protein is to build proteins in the body, many amino acids have an important role in the sow’s metabolism around farrowing when insulin sensitivity is low: they provide glucogenic substrate to the citric acid cycle [24]. The practical relevance of an improved nutrient utilization in the case of lysolecithin should be reflected in litter growth during lactation. Indeed, litter weight at weaning was higher in the LLG group. This finding agrees with those of Wang et al. [21], who reported improved litter weight gain in the lysophospholipid supplemented group. Similarly, supplementation of soybean lecithin in sow diets during gestation and lactation resulted in improved piglet weight at weaning [23].

In the study of Zhao et al. [20], the addition of LL in beef tallow-containing sow diets led to a significant reduction in body weight and backfat loss between farrowing and weaning due to increased digestibility of dry matter, nitrogen, gross energy and crude fat. Due to the different measurement schedule, though, it is difficult to assess the exact point in lactation when the positive effect of LL becomes more prominent. It must be stressed that a direct comparison with our results must be carried out cautiously, since no animal fats were included in the feed and, as aforementioned, a significant interaction between LL activity and fat type is to be expected.

The positive results observed on piglet weight at weaning could also be attributed to possible effects on composition of sow milk and colostrum. Zhao et al. [20] reported that the addition of LL in beef tallow-containing sow diets can lead to an increase in the fat and lactose content of the milk, depending on the stage of lactation. Wang et al. [21] also reported a positive effect of LL supplementation on IgG/A/M levels in sow colostrum and milk and on IgA/M levels in piglet blood. An increase in immunoglobulin (mainly IgA) content of the colostrum, milk, sow blood and suckling piglet blood was confirmed by Shi et al. [23] who, furthermore, found a respective increase in the levels of phosphatidylcholine and total PLs and a significantly decreased diameter of fat globules in the milk of sows fed on a lecithin-enforced diet. Therefore, it is plausible that the increased litter weight at weaning in our study could be attributed to an elevated energy/nutrient content of the consumed milk, combined with its increased digestibility and immunoglobulin levels, offering passive immunity and resilience to bacterial and viral pathogens and to food antigens [23].

Glucose is the main energy source for both the sow and her litter [4,25]. Its homeostasis balances on the dietary carbohydrate intake, the metabolic needs and a set of hormones (e.g., insulin and glucagon) and organs (e.g., kidneys, liver) that regulate its utilization, storage and elimination. According to our results, the preprandial glucose concentrations on lactation day 14 were lower in the animals of the LLG group, although the difference with the CG group did not reach statistical significance. This could generally indicate either a reduction in the assimilation of feed nutrients or a better peripheral utilization. Lower blood glucose levels in sows have been associated with an improved insulin sensitivity [24]. Wang et al. [21] reported increased blood glucose levels due to lysophospholipid supplementation. Nevertheless, in the latter study blood parameters were evaluated at approximately 3 h postprandially and cannot be fully compared to our results. According to Laws et al. [22] dietary fat supplementation in sows can result in elevated glucose concentrations because of a fat-induced glucose intolerance. In our study we have used commercially relevant fat levels, nearly half the supplemented quantity of those used by Laws et al. [22]. Presumably if fat levels were higher than the current ones, the differences in the pre- and postprandial levels of glucose and triglycerides may have become significant. Furthermore, the delayed interval for the peak value of triglyceride concentration in the LLG group both at day 110 of gestation and at day 14 of lactation, may indicate a prolonged digestion and absorption procedure of dietary lipids due to a more efficient emulsification procedure and breakdown to smaller micelles by the action of lysolecithin. This field warrants further investigation.

A promising finding was detected for postprandial urea levels during lactation reflecting the use of amino acids as energy source, which were significantly higher in the LLG sows. We postulate that the addition of LL in the feed results in a trend for more sustained absorption and systematic delivery at least of proteins at a rate which allows for a better peripheral utilization. This effect could be attributed to the overall improvement of crude protein digestibility along with other nutrients, as demonstrated by Wang et al. [21] in the case of lysophospholipid supplementation.

Leptin is a hormone produced by adipocytes. It acts as a regulator of energy homeostasis and serves as an indicator of body condition since its levels reflect body fat stores [2,24]. On gestation day 108, significantly higher leptin concentration in the blood of sows of group CG were probably correlated with increased backfat thickness. On lactation day 14, although backfat at the P2 position was still significantly thicker in the CG group, the divergence was probably beginning to drop enough (due to the decrease in the fat loss rate in the animals of the LLG group), so that the respective difference in leptin concentrations was not deemed significant. Nevertheless, the physiological significance of the higher leptin levels in the CG group may be related to a post-metabolic effect on the growth of the progeny. It was previously shown that significant negative correlations exist between ante-partum leptin levels and litter weight and litter growth during lactation [26]. Interestingly, in the CG group the higher leptin levels ante-partum co-existed with the lower litter weight at weaning.

## 5. Conclusions

In conclusion, lysolecithin supplementation in sows resulted in improved litter weight at weaning without an excessive catabolism of backfat tissue during the mid-lactation period, most probably due to an efficient nutrient utilization, which warrants further investigation.

## Figures and Tables

**Table 1:** Main ingredients (in kg/1000 kg) and nutrients (in %) of the gestation and lactation diet.

**Ingredients**	**Gestation Diet**	**Lactation Diet**
Corn	317.5	289.0
Barley	200.0	150.0
Wheat Bran	300.0	270.0
DDGS Corn	50.0	50.0
Soybean, 44% CP	75.0	140.0
Fishmeal	-	20.0
Animal Fat	20.0	40.0
Limestone	14.0	14.5
Monocalciumphosphate	6.0	8.0
Lysine HCl	2.0	2.0
L- threonine	0.5	0.5
Sodium Chloride	5.0	6.0
Premix ^a^	10.0	10.0
Nutrients ^b^	**Gestation Diet**	**Lactation Diet**
NE (Kcal/kg)	2311	2387
Crude protein	13.75	16.65
Crude fat	5.25	7.27
Crude fiber	4.33	4.24
Ash	5.17	5.92
Starch	37.15	32.13
Ca	0.69	0.84
P	0.61	0.70
Amino acids-Standarized ileal digestibility		
Lysine	0.60	0.80
Meth. + Cyst.	0.39	0.47
Threonine	0.42	0.53
Tryptophane	0.12	0.16
Isoleucine	0.41	0.54
Valine	0.53	0.66

^a^ Provided per kg diet: Vitamin A 12,000 IU; Vitamin D3 2000 IU; Vitamin E 60 mg; Vitamin K3 3 mg; Vitamin B1 1.5 mg; Vitamin B2 5 mg; Vitamin B6 5 mg; Vitamin B12 0.025 mg; folic acid 2 mg; nicotinic acid 30 mg; biotin 0.25 mg; D-pantothenic acid 15 mg; Fe 100 mg, Cu 15 mg, Mn 50 mg, I 2 mg, Se 0.3 mg; ^b^ Calculated based on “Centraal Veevoederbureau (CVB) Feed Table 2018-Chemical composition and nutritional values of feedstuffs” (available at: www.cvbdiervoeding.nl, accessed on 3 November 2021) and using the software Plurimix (Fabermatica, Piazza Bruno Pari 3–26032 Ostiano (CR), Italy).

**Table 2:** Reproductive performance parameters of sows according to treatment (*n* = 30 per treatment).

Parameter	Treatment		SEM	*p*
	CG	LLG		
Parity	4.4	4.8	0.39	0.512
Body Weight (kg)				
day 108 gestation	291.3	281.6	5.51	0.217
weaning	215.9	215.4	4.65	0.946
weight loss	75.4	67.5	3.54	0.123
weight loss (%)	25.5	23.1	1.01	0.092
Backfat (mm)				
day 108 of gestation	21.8	18.9	0.91	0.030
day 14 lactation	16.0	13.9	0.72	0.044
loss d108-d14	5.8	5.0	0.52	0.298
loss d108-d14 (%)	25.4	25.7	1.93	0.923
weaning	13.8	12.2	0.65	0.140
loss d108-weaning	8.0	6.7	0.58	0.124
loss d108-weaning (%)	35.4	34.8	2.07	0.847
loss d14-weaning	3.1	1.9	0.32	0.006
loss d14-weaning (%)	18.6	13.7	2.01	0.079
Weaning to estrus interval (days)	5.8	6.0	0.67	0.834

**Table 3:** Effects of treatments on piglet performance parameters (*n* = 30 per treatment).

Parameter	Treatment		SEM	*p* Value
	CG	LLG		
Liveborn	12.5	13.1	0.39	0.292
Weaned	11.3	11.8	0.21	0.103
Died during lactation	1.3	1.4	0.18	0.707
Litter weight at birth (kg)	17.4	18.0	0.48	0.381
Litter weight at weaning (kg)	84.7	89.8	1.61	0.027
Average piglet weight at birth (g)	1447.7	1431.6	33.28	0.734
Coefficient of variation of piglet weight at birth (%)	19.9	19.1	1.01	0.564
Average piglet weight at weaning (g)	7587.6	7679.6	152.24	0.671
Coefficient of variation of piglet weight at weaning (%)	14.7	15.5	0.82	0.533

**Table 4:** Average blood glucose levels of sows before and 30, 60, 90 and 120 minutes after the consumption of morning meal according to treatment (*n* = 15 per treatment).

Glucose Level (mg/dL)	Treatment		*p*
	CG	LLG	
Gestation day 108			
Baseline	48.3 ± 9.23	49.8 ± 10.51	0.858
30 min post-prandial	61.3 ± 15.68	67.7 ± 16.38	0.476
60 min post-prandial	56.3 ± 8.38	54.5 ± 5.88	0.594
90 min post-prandial	48.8 ± 9.70	53.1 ± 9.47	0.906
120 min post-prandial	53.3 ± 10.14	59.5 ± 12.3	0.674
Lactation day 14			
Before meal consumption	48.8 ± 11.96	38.4 ± 15.50	0.074
30 min post-prandial	66.8 ± 10.69	70.0 ± 10.49	0.475
60 min post-prandial	66.8 ± 14.43	67.3 ± 11.94	0.689
90 min post-prandial	62.5 ± 14.27	60.6 ± 7.09	0.656
120 min post-prandial	71.4 ± 17.22	66.5 ± 9.50	0.450

**Table 5:** Average blood triglycerides level (mg/dL) of sows before and 30, 60, 90 and 120 min after the consumption of morning meal, according to treatment (*n* = 15 per treatment).

Triglycerides Level (mg/dL)	Treatment		*p*
	CG	LLG	
Gestation day 108			
Baseline	79.3 ± 19.29	73.5 ± 6.16	0.317
30 min post-prandial	82.4 ± 17.10	85.7 ± 35.21	0.500
60 min post-prandial	85.5 ± 23.56	80.2 ± 16.82	0.500
90 min post-prandial	82.4 ± 17.64	83.6 ± 17.48	NA ^1^ (1.000)
120 min post-prandial	85.0 ± 22.11	81.5 ± 21.55	0.080
Lactation day 14			
Before meal consumption	86.4 ± 31.03	79.3 ± 19.28	0.285
30 min post-prandial	79.2 ± 22.79	71.3 ± 2.69	0.225
60 min post-prandial	73.5 ± 7.92	95.5 ± 56.8	0.109
90 min post-prandial	74.9 ± 9.56	75.2 ± 9.83	0.655
120 min post-prandial	83.3 ± 30.46	81.2 ± 23.75	0.588

NA ^1^: Not available.

**Table 6:** Blood parameters (mean ± standard deviation) on day 108 of gestation and on day 14 of lactation (*n* = 15 per treatment).

Parameter	Gestation Day 108			Lactation Day 14		
	CG	LLG	*p*	CG	LLG	*p*
Urea (mg/dL)	28.7 ± 5.50	27.8 ± 3.74	0.538	23.5 ± 6.23	30.3 ± 5.61	0.002
NEFA (mmol/L)	0.17 ± 0.076	0.19 ± 0.079	0.544	0.18 ± 0.102	0.23 ± 0.114	0.253
Creatinine (mg/dL)	1.8 ± 0.22	1.8 ± 0.23	0.816	2.1 ± 0.27	2.0 ± 0.17	0.642
Leptin (ng/mL)	3.7 ± 3.65	1.2 ± 1.32	0.021	6.8 ± 7.07	5.8 ± 5.68	0.381

## Data Availability

Data of the study are available upon request.
